# M2 macrophages-derived exosomes for osteonecrosis of femoral head treatment: modulating neutrophil extracellular traps formation and endothelial phenotype transition

**DOI:** 10.1038/s41413-025-00412-5

**Published:** 2025-04-01

**Authors:** Guanzhi Liu, Ruomu Cao, Qimeng Liu, Heng Li, Peng Yan, Kunzheng Wang, Run Tian, Pei Yang

**Affiliations:** 1 Department of Bone and Joint Surgery, The Second Affiliated Hospital of Xi’an Jiaotong University, Xi’an, China; 2 Department of Orthopedics, The First Affiliated Hospital, Zhejiang University School of Medicine, Hangzhou, China

**Keywords:** Bone, Pathogenesis

## Abstract

Exosomes have shown good potential in ischemic injury disease treatments. However, evidence about their effect and molecular mechanisms in osteonecrosis of femoral head (ONFH) treatment is still limited. Here, we revealed the cell biology characters of ONFH osteonecrosis area bone tissue in single cell scale and thus identified a novel ONFH treatment approach based on M2 macrophages-derived exosomes (M2-Exos). We further show that M2-Exos are highly effective in the treatment of ONFH by modulating the phenotypes communication between neutrophil and endothelium including neutrophil extracellular traps formation and endothelial phenotype transition. Additionally, we identified that M2-Exos’ therapeutic effect is attributed to the high content of miR-93-5p and constructed miR-93-5p overexpression model in vitro and in vivo based on lentivirus and adeno-associated virus respectively. Then we found miR-93-5p can not only reduce neutrophil extracellular traps formation but also improve angiogenic ability of endothelial cells. These results provided a new theoretical basis for the clinical application of ONFH therapeutic exosomes.

## Introduction

Osteonecrosis of the femoral head (ONFH) is a painful, refractory, and debilitating bone disease characterized by the gradual death of bone cells and bone marrow cells.^1,2^ This pathological process ultimately results in structural changes or collapse of the femoral head and destruction of the hip joint.^3–5^ Single-cell RNA sequencing (scRNA-seq) is a valuable technique for investigating the cellular status in bone and bone marrow.^6–8^ Nevertheless, the precise pathogenesis of ONFH based on scRNA-seq method remains poorly understood.

Recently, immune regulation and the immune environment of bone tissue have attracted attention.^9,10^ Bone homeostasis can be adversely affected by abnormal immune function, and pathological changes in bone tissue can also have a significant impact on immune phenotype.^11,12^ Neutrophils play a crucial role in bone immune regulation, with one of the most important phenotypes being the occurrence of NETosis and the release of neutrophil extracellular traps (NETs) composed of histones, granzymes, and nuclear components.^13,14^ There is increasing evidence from recent years that NETs are also involved in immune regulation of diseases like vasculitis, thrombosis, and trauma.^15,16^ However, immune regulation by neutrophils in ONFH bone tissue and its effects on the phenotype of ONFH endothelial cells have remained unclear. At present, single-cell level experiments provide very limited insight into neutrophil immune regulation related to ONFH. Evidences about the potential role and molecular biology mechanisms of NETs in ONFH are urgently needed to be addressed.

Endothelial to mesenchymal transition (EndoMT) occurs when endothelial cells migrate away from the endothelium with the reduce of endothelial characteristics and the increase of mesenchymal characteristics.^17,18^ This process has been proven to affect endothelial function and participate in various physiological and pathological processes.^19,20^ Additionally, the formation of neutrophil NETs will promote the initiation of EndoMT in endothelial cells.^21,22^ To date, evidence is lacking on whether NETs induced EndoMT is involved in the development of ONFH.

Both prokaryotic and eukaryotic cells produce extracellular vesicles (EVs), which are membrane-enclosed nanoparticles that facilitate intracellular communication by transferring bioactive molecules from donor cells to recipient cells selectively.^23,24^ In pathology and physiology, M2 macrophages polarization plays an important role in anti-inflammatory processes and immune regulation.^25,26^ M2 macrophage derived exosomes (M2-Exos) have attracted attention, and some studies have found that they can induce oxidative stress and regulate inflammation.^27,28^ Qian et al. suggested that M2-Exos miR-26b-5p regulates macrophage polarization and chondrocyte hypertrophy by targeting TLR3 and COL10A1. Wang et al. demonstrated that M2-Exos attenuated LPS-induced cell apoptosis by regulating the miR-93-5p/TLR4 axis.^29,30^ However, M2-Exos’ specific role in the occurrence and development of ONFH is not yet clear.

Hence, this study analysed femoral head osteonecrosis area cells from ONFH patients and identified the key cell cluster and their communication status. Next, we developed corresponding ONFH treatment strategy based on M2-Exos and then explored the therapeutic effect and molecular mechanism of M2-Exos from a new perspective of NETs regulation.

## Results

### Single-cell transcriptome analysis of ONFH subchondral bone tissue

**Fig. 1:**
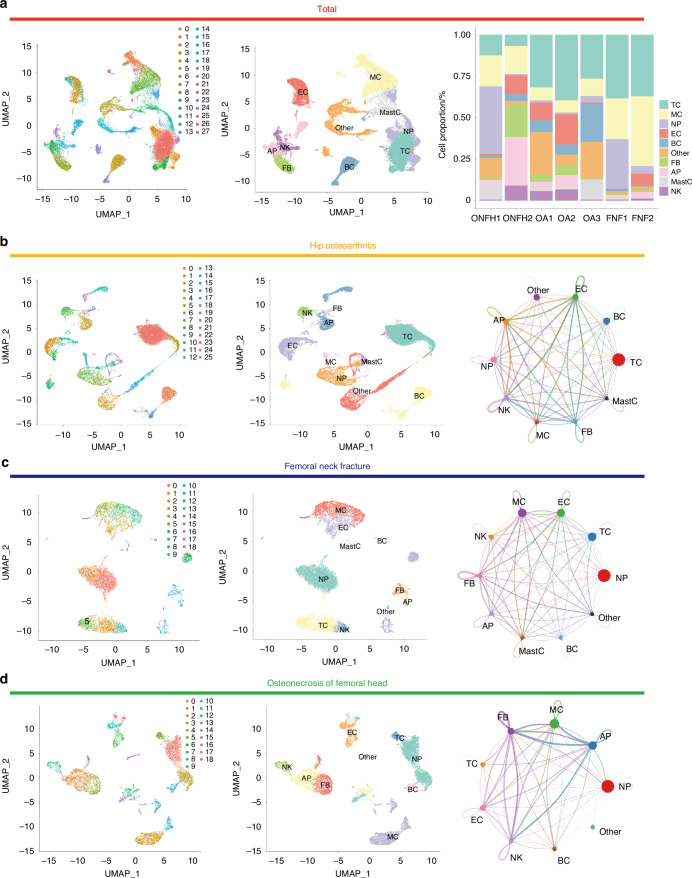
Single-cell transcriptome analysis of ONFH subchondral bone tissue. **a** ScRNA sequencing cell cluster and annotation analysis of total samples (ONFH *n* = 2; FHF *n* = 2; hip osteoarthritis *n* = 3). **b** HOA group cell cluster and cell-cell communication analysis. **c** FHF group cell cluster and cell-cell communication analysis. **d** ONFH group cell cluster and cell-cell communication analysis

To decipher the key cell clusters and phenotypes in ONFH, scRNA-seq analysis on subchondral bone tissues were performed (ONFH *n* = 2; femoral neck fracture *n* = 2; hip osteoarthritis *n* = 3). After initial stringent quality control and doublet removal, a total of 55 981 individual cells were profiled. Median UMI counts per cell was 6 190 and median genes per cell was 2 057. Based on t-SNE analysis, unbiased clustering of the cells parallelly identified 10 main clusters according to their gene profiles and canonical markers (Fig. 1a). Next, we carried out t-SNE analyses and cell-cell communication analyses in ONFH, FHF and HOA group respectively. Results indicated that neutrophil seemed to play important roles in cell communications in all these group. ONFH bulk RNA-seq cellular composition analyses also validated these results (Fig. S1). Further ligand-receptor interaction analyses showed that the VEGFA, VEGFB and CXCL8 associated interaction pairs were significant in the crosstalk between neutrophil and endothelium in hip osteoarthritis (HOA) group which may enhance the angiogenesis. Meanwhile, the increasing of TGFB associated interactions in the crosstalk between neutrophil and fibroblasts were also observed in HOA group. Femoral neck fracture (FHF) group showed the raise of VEGFA associated interactions in the crosstalk between neutrophil and endothelium, while the TGFB associated interaction strength in the crosstalk between neutrophil and fibroblasts was not significant. Notably, TGFB associated interaction pairs played important roles in the crosstalk between neutrophil and fibroblasts in ONFH group, while angiogenesis associated interactions in the crosstalk between neutrophil and endothelium were limited (Fig. 1b–d, Figs. S2–7).

### Activation of NETs formation in ONFH rat femoral head

**Fig. 2:**
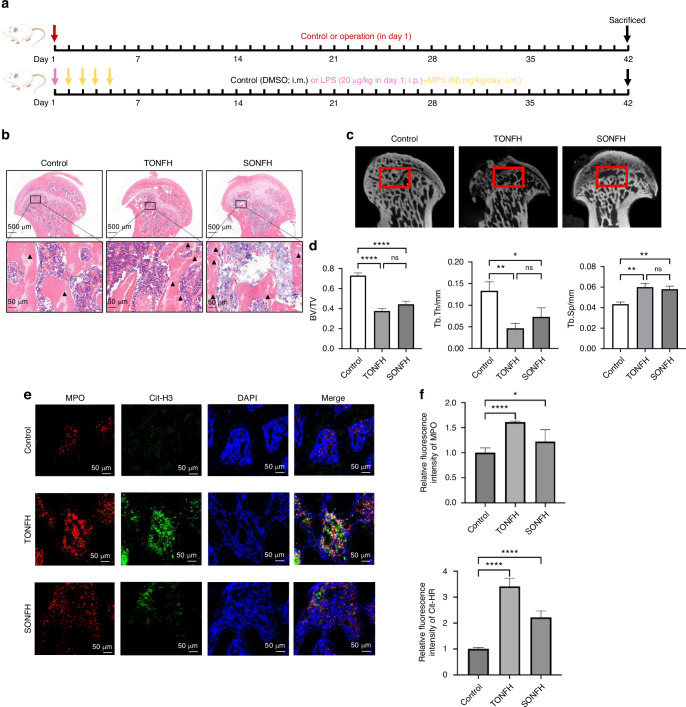
ONFH rat model construction and NETs formation in femoral head. **a** TONFH rat models and SONFH rat models establishment pipelines. **b** HE stanning of rat femoral heads in control group, TONFH group and SONFH group. **c**, **d** MicroCT analysis of rat femoral heads in control group, TONFH group and SONFH group. **e**, **f** Immunofluorescent staining of NETs (MPO: red; Cit-H3: green; DAPI: blue). All data were presented as means ± SD, *n* ≥ 3 per group; ns *P* ≥ 0.05. *P < 0.05. **P < 0.01. ***P < 0.001. ****P < 0.000 1; Statistical significance was determined by two-tailed Student’s *t* test (**d**, **f**).Triangle marker: area with empty osteocytic lacunae

To determine the ONFH-associated phenotypes of neutrophil and endothelial cell in the femoral head, we constructed trauma-induced osteonecrosis of femoral head (TONFH) rat models and steroid-induced osteonecrosis of femoral head (SONFH) rat models using the modeling pipelines in Fig. 2a. Hematoxylin and eosin (H&E) staining showed the signs of osteonecrosis among these groups. First, the trabecular bone and marrow structures were profoundly destroyed or even completely eliminated in the femoral heads of both TONFH group and SONFH group. Second, the empty osteocytic lacunae were detected in the residual trabecular bones of the femoral heads of TONFH group and SONFH group but were rarely observed in normal group (Fig. 2b). Micro-computed tomography (μCT) analysis showed that TONFH group and SONFH group have more low-density area (osteonecrotic lesion) and remarkable bone loss in the femoral heads. Meanwhile, we also detected the markedly decreased trabecular bone volume fraction (BV/TV), trabecular thickness (Tb. Th), and the notably increased trabecular separation (Tb. Sp) in the TONFH group and SONFH group compared with the normal group (Fig. 2c, d). Specifically, the neutrophil NETs formation was identified by immunofluorescence staining based on the molecular markers MPO (neutrophil marker), Cit-H3 (citrullinated histone marker) and DAPI (DNA marker). The results of our study showed that TONFH group and SONFH group have higher expression of MPO and Cit-H3. NETs released by neutrophils in subchondral bone of TONFH rat model and SONFH rat model were confirmed by the colocalization of MPO, Cit-H3 and DNA (Fig. 2e, f).

### M2-Exos enhance HUVECs angiogenesis by reducing NETs formation and endothelial phenotype transition in vitro

**Fig. 3:**
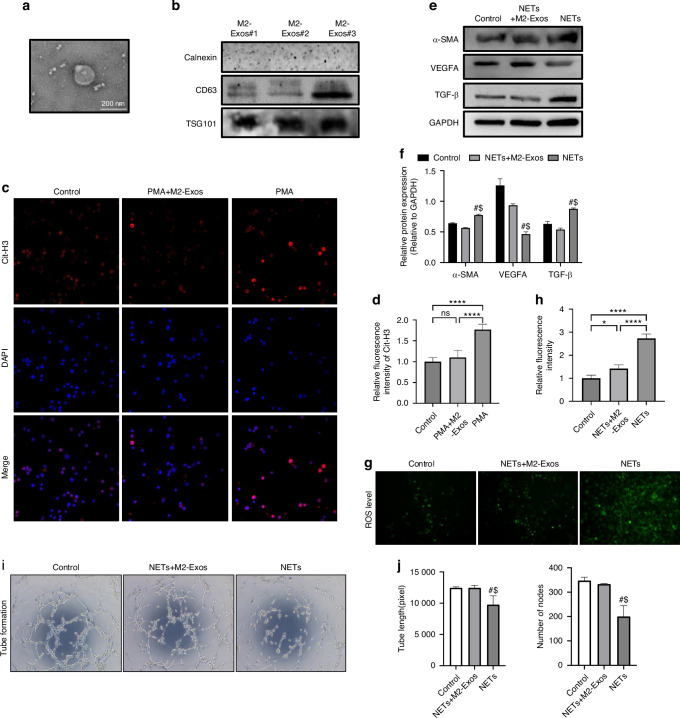
M2-Exos enhance HUVECs angiogenesis by reducing NETs formation and endothelial phenotype transition in vitro. **a** Transmission electron microscopy (TEM) image of M2-Exos. **b** Western blotting assay for specific markers of exosomes like CD63, Tsg101 and endoplasmic reticulum protein Calnexin. **c**, **d** In vitro immunofluorescent staining for NETs formation after the treatment of PMA and M2-Exos (Cit-H3: red; DAPI: blue). **e**, **f** Western blotting assay for angiogenesis markers and mesenchymal markers in HUVECs after the treatment of NETs and M2-Exos. **g**–**j** HUVECs ROS levels and tube formation ability after the treatment of NETs and M2-Exos. All data were presented as means ± SD, *n* ≥ 3 per group; ns *P* ≥ 0.05. *P < 0.05. **P < 0.01. ***P < 0.001. ****P < 0.000 1; In (**f**, **j**), # represents the differences between NETs group and Control group have statistical significance (*P* < 0.05); $ represents the differences between NETs+M2-Exos group and NETs group have statistical significance (*P* < 0.05). Statistical significance was determined by two-tailed Student’s *t* test (**d**, **f**, **j**, **h**)

Macrophage culture, M2 polarization, exosomes isolation and purification were carried out according to our previous research.^31^ Then, exosomes were verified by transmission electron microscopy (TEM) and morphologically M2-Exos were cup‐shaped spherical vesicles in TEM images (Fig. 3a). Western blotting showed positive expression of specific markers of exosomes like CD63 and Tsg101, while negative expression of endoplasmic reticulum protein Calnexin (Fig. 3b). Immunofluorescence staining of Cit-H3 showed that PMA treatment markedly increased the NET formation, which can be significantly reduced by M2-Exos (Fig. 3c, d). In addition, we used purified NETs in vitro to incubate with HUVECs and performed western blot assays. The results suggested that the expression of mesenchymal makers α-SMA significantly increased by the treatment of NETs in HUVECs, while the expression of angiogenesis marker VEGFA were markedly reduced. The expression of TGF-β was also significantly increased which commonly considered as the primary inducer of endothelial phenotype transition (Fig. 3e, f). Further ROS level detection and tube formation assays showed NETs treatment significantly increased the ROS levels and decreased the tube formation compared with control group, which can be rescued with the addition of M2-Exos (Fig. 3g–j). These results indicated that NETs treatment caused HUVECs angiogenesis dysfunction by promoting endothelial phenotype transition and further perivascular ECM remodeling. Notedly, the addition of M2-Exos significantly yield an apparent reduction of endothelial phenotype transition and increased VEGFA expression in NETs-treated HUVECs.

### M2-Exos improve ONFH by reducing NETs formation in vivo

**Fig. 4:**
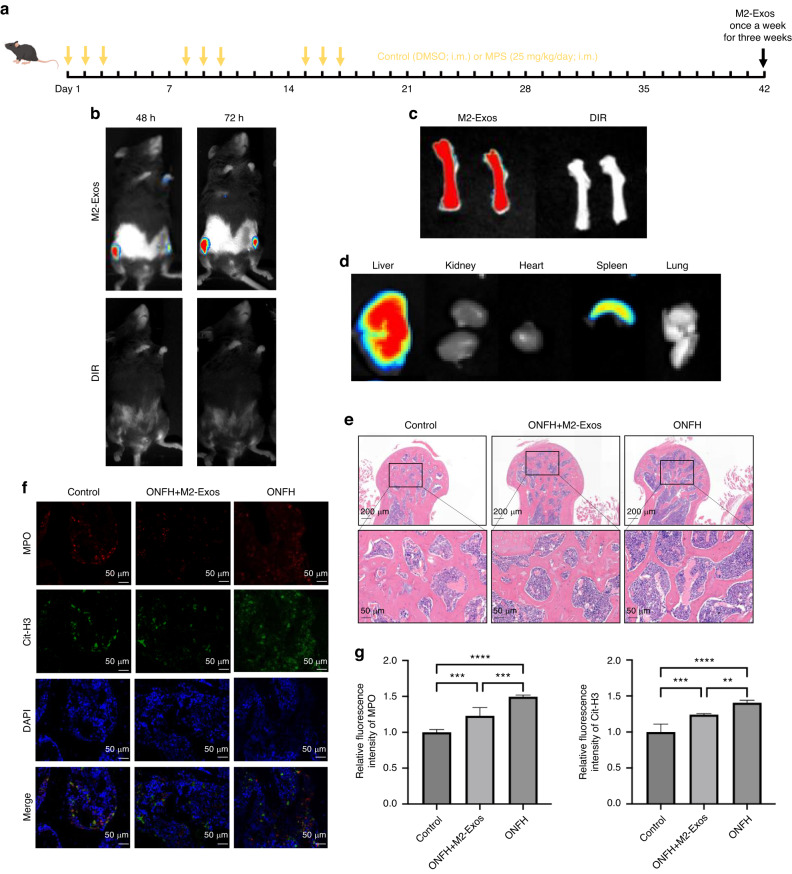
M2-Exos improve ONFH by reducing NETs formation in vivo. **a** SONFH mice model establishment and M2-Exos injection pipeline. **b** Representative fluorescence images after intravenous injections of DIR (control group) and DIR-labeled M2-Exos for 48 h and 72 h. **c** Representative fluorescence images of the distribution of M2-Exos in the femur after 72 h. **d** Representative fluorescence images of the distribution of M2-Exos in the main organs after 72 h. **e** HE stanning of SONFH mice femoral heads in control group, SONFH + M2-Exos group and SONFH group. **f**, **g** Immunofluorescent staining for NETs formation in SONFH mice femoral head (MPO: red; Cit-H3: green; DAPI: blue). All data were presented as means ± SD, *n* ≥ 3 per group; ns *P* ≥ 0.05. *P < 0.05. **P < 0.01. ***P < 0.001. ****P < 0.000 1; Statistical significance was determined by two-tailed Student’s *t* test (**g**)

We first detect the targeting ability of M2-Exos in the femoral head of SONFH mice model using an in vivo imaging system. M2-Exos were labeled with fluorescent dye DIR and then injected into mice through a tail vein (Fig. 4a). M2-Exos distribution was imaged at 24, 48 and 72 h time points. Results showed the both M2-Exos group and control group (DIR dye) have weak fluorescence signals in femoral head at 24 h. However, the fluorescence intensity in femoral head of M2-Exos group gradually increased at 48 h and 72 h, which indicated the excellent ONFH targeting ability of M2-Exos (Fig. 4b). 72 h later, the mice were sacrificed and the femur and main organs were removed completely to perform the fluorescence intensity evaluation. Results showed significant fluorescence signals in femur of M2-Exos group compared to control group (Fig. 4c). Further analysis of the fluorescence intensity of major organs showed that the fluorescence signals of exosomes were mainly concentrated in the liver and spleen due to the clearance of the mononuclear macrophage system (Fig. 4d). Next, to investigate the therapeutic effects of M2-Exos on ONFH, SONFH mice model was established and received intravenous injections of M2-Exos (200 µg) once a week for three weeks. After the intervention, the mice were euthanized, and the femur were excised for HE stanning. ONFH group exhibited destruction of trabecular bone and marrow structures and higher empty osteocytic lacunae rate were observed in the residual trabecular bones of the femoral heads of ONFH group than control group. Compared to the ONFH group, M2-Exos treatment rehabilitate trabecular bone and marrow structures and reduce the empty osteocytic lacunae rate (Fig. 4e). Additionally, NETs immunofluorescence staining assay showed ONFH mice have higher expression of MPO and Cit-H3 which can be reduced by the treatment of M2-Exos. These results suggested that M2-Exos have excellent targeting ability and can improve ONFH by modulating NETs formation in vivo (Fig. 4f, g).

### M2-Exos miR-93-5p regulate NETs-HUVECs interaction and endothelial phenotype transition activation in vitro

**Fig. 5:**
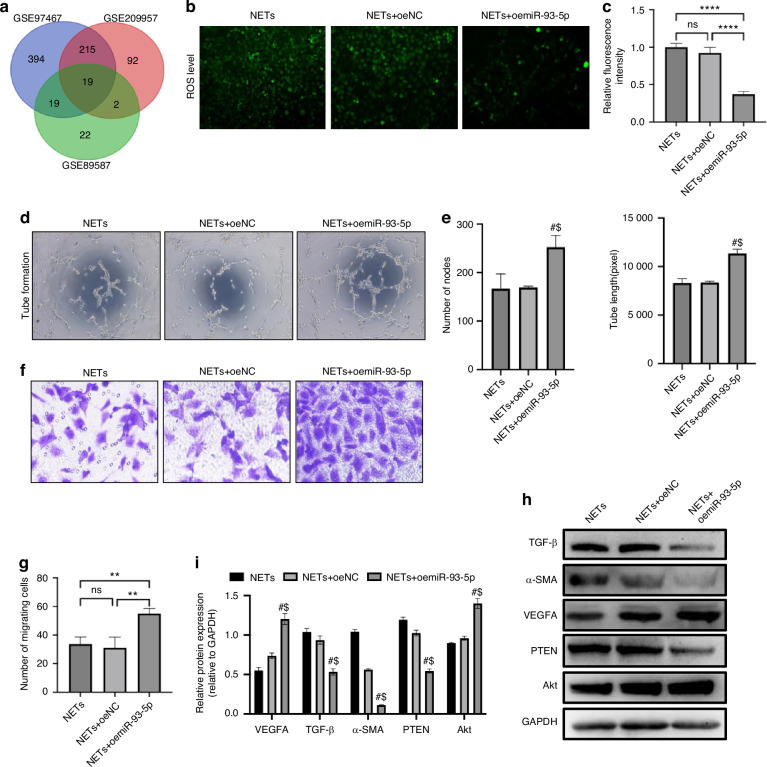
M2-Exos miR-93-5p regulate NETs-HUVECs interaction and endothelial phenotype transition activation in vitro. **a** ONFH associated M2-Exos miRNA analysis (GSE97467: M2-Exos miRNA dataset 1; GSE209957: M2-Exos miRNA dataset 2; GSE89587: ONFH patients serum deferential expressed miRNAs compared to control group). **b**–**e** ROS levels and tube formation ability after the treatment of NETs in HUVECs group, HUVECs overexpression control group and HUVECs miR-93-5p overexpression group. **f**, **g** HUVEC transwell migration assays. **h**, **i** Western blotting assay for angiogenesis markers and mesenchymal markers in each HUVEC group. All data were presented as means ± SD, *n* ≥ 3 per group; ns *P* ≥ 0.05. *P < 0.05. **P < 0.01. ***P < 0.001. ****P < 0.000 1; In (**e**, **i**), # represents the differences between NETs+oemiR-93-5p group and NETs group have statistical significance (*P* < 0.05); $ represents the differences between NETs+oemiR-93-5p group and NETs+oeNC group have statistical significance (*P* < 0.05). Statistical significance was determined by two-tailed Student’s *t* test (**c**, **e**, **f**, **g**–**i**)

In order to explore the molecular mechanism of M2-Exos to prevent ONFH development. combination analyses were performed to investigate the hub miRNA associated with ONFH in M2-Exos. Results showed 19 M2-Exos miRNAs have high correlation with ONFH (Fig. 5a). We further focused on miR-93-5p and constructed miR-93-5p over-expression HUVECs model using lentivirus (Fig. S8). Results showed miR-93-5p over-expression HUVECs have significantly lower ROS levels and more migration and tube formation compared with NETs treated group and lentivirus-NC treated group (Fig. 5b–g). In addition, western blot assays suggested that the expression of mesenchymal makers α-SMA, primary inducer marker of endothelial phenotype transition TGF-β and PTEN significantly reduced by the over-expression of miR-93-5p. While the expression angiogenesis effect factor VEGFA and Akt significantly increased with the over-expression of miR-93-5p (Fig. 5h, i). These results suggested that miR-93-5p can regulate the PTEN/Akt axis and involved with the HUVECs angiogenesis function by mediating the NETs-HUVECs interaction and endothelial phenotype transition activation.

### MiR-93-5p is involved with the therapeutic effects of M2-Exos for ONFH by prohibiting NETs formation

**Fig. 6:**
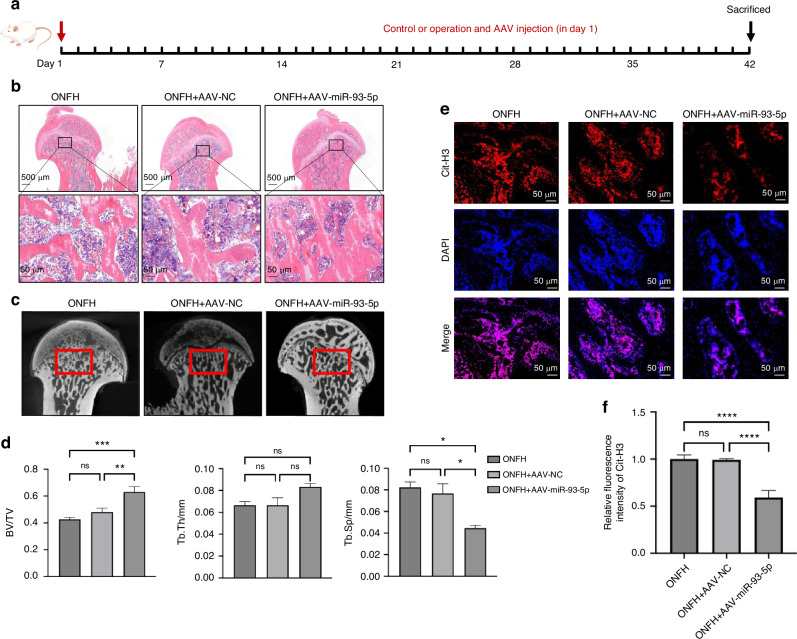
MiR-93-5p is involved with the therapeutic effects of M2-Exos for ONFH by prohibiting NETs formation. **a** ONFH rat model adeno-associated virus (AAV) treatment pipeline. **b** HE stanning of ONFH rat femoral heads in ONFH group, AAV negative control group and AAV-miR-93-5p group. **c**, **d** MicroCT images and structure analyses of rat femoral heads. **e**, **f** Immunofluorescent staining for NETs formation in rats’ femoral head (Cit-H3: red; DAPI: blue). All data were presented as means ± SD, *n* ≥ 3 per group; ns *P* ≥ 0.05. *P < 0.05. **P < 0.01. ***P < 0.001. ****P < 0.000 1; Statistical significance was determined by two-tailed Student’s *t* test (**d**, **f**)

To further investigate the involvement of miR-93-5p for ONFH treatment in vivo, the AAV-miR-93-5p ONFH rat model was established (Fig. 6a). AAV9 vector transduction situation in bone tissue and period of validity are shown in Fig. S9. After 6 weeks of modeling, the rats were sacrificed and the femurs were removed for HE stanning. Results showed plenty of empty osteocytic lacunae was observed in ONFH group and AAV-NC group, which also exhibited destruction of trabecular bone and marrow structures. AAV-miR-93-5p treatment group exhibited much milder trabecular bone and marrow destruction in the femoral heads compared with the ONFH group and AAV-NC group (Fig. 6b). Micro-computed tomography (μCT) analysis also showed that ONFH group and AAV-NC group have more osteonecrotic area and remarkable bone loss in the femoral heads. AAV-miR-93-5p treatment yield an apparent reduction of trabecular bone and marrow destruction. Meanwhile, we also detected the markedly increased trabecular bone volume fraction (BV/TV), trabecular thickness (Tb. Th), and the notably reduced trabecular separation (Tb. Sp) in the AAV-miR-93-5p group compared to ONFH group and AAV-NC group (Fig. 6c, d). In addition, we detected the NETs formation in femoral head of these groups via Immunofluorescence staining of Cit-H3. Results showed that rats with AAV-miR-93-5p treated have significantly lower NETs level than ONFH group and AAV-NC group (Fig. 6e, f), which indicated that miR-93-5p may prevent ONFH development by prohibiting NETs formation in ONFH rat femoral head. The therapeutic effects of M2-Exos may attribute to the NETs regulation function of miR-93-5p.

## Discussion

Osteonecrosis of the femoral head (ONFH) is a prevalent and debilitating orthopedic disease.^32,33^ Dysregulation of immune function can profoundly influence bone homeostasis, potentially exacerbating the development of ONFH.^34–36^ Our research has identified neutrophil activation and the formation of neutrophil extracellular traps (NETs) as key drivers of ONFH pathogenesis. Strikingly, there is a notable lack of comprehensive studies investigating effective therapeutic interventions targeting NETs formation for the treatment of ONFH. Hence, we focused our study on the phenotype and molecular mechanisms of M2-Exos, which could target to bone tissue including the femoral head and prevent ONFH development by regulating NETs formation and improve angiogenesis in osteonecrosis area. The development of such strategies could revolutionize current treatment approaches and offer significant clinical advantages.

Previous researches on ONFH have primarily examined the canonical phenotype like osteogenic and angiogenic.^37,38^ However, there remains a dearth of single-cell scale evidence elucidating the specific cell clusters that play crucial roles in ONFH.^39,40^ Our single cell sequencing data revealed the categorization and annotation of ONFH cells within the osteonecrosis area into 10 distinct subpopulations. Further cell communication analyses revealed that neutrophil can play important roles in ONFH development. The communication characteristics between neutrophil and endothelium cells were highly different in ONFH group compared with OA group and FHF group. Neutrophils can respond to DMAPs stimulation and secrete cytokines to regulate the inflammatory immune environment.^41,42^ It has been proven that it can recruit BMSCs to promote osteogenesis through SDF-1/CXCR4 axis.^13^ Inducing neutrophils to produce NETs can inhibit osteoblast activity and enhance osteoclast activity.^43^ Neutrophil can also participate in regulating biological processes such as vascular permeability and angiogenesis via CXCL8 pathway.^44^ An observational study has shown that indicators such as neutrophil count and neutrophil lymphocyte ratio are significantly correlated with the occurrence of femoral head necrosis, but the specific molecular mechanism is not yet clear.^45^ EndoMT is a process that an endothelial cell undergoes a series of molecular events that lead to a change in phenotype toward a mesenchymal cell.^46,47^ It has been found to participate in the pathophysiological processes of atherosclerosis, diabetes nephropathy and other diseases.^22,48^ However, whether EndoMT occurs in ONFH has not been effectively demonstrated. In addition, Recent research has also identified that the formation of NETs facilitates the initiation of EndoMT in endothelial cells, ultimately impairing endothelial function. Hence, investigating these multifaceted interplays between NETs and EndoMT has the potential to yield transformative insights and therapeutic avenues in the realm of ONFH treatment. Some studies have demonstrated stem cell-based therapy have considerable potential in the treatment of ONFH.^49^ However, some drawbacks like tumorigenic potential, immunogenicity and ethical issue limited its clinical application.^50^ Thus, with possess numerous advantages such as non-immunogenicity, non-infusion toxicity, easy access and effortless preservation, stem cell-derived exosomes now being extensively exploited to develop novel therapeutic strategies for ONFH.^51,52^ Nevertheless, currently researches focused on some other types of exosome-based therapy for ONFH was limited. M2-Exos, which inherit surface constituents such as proteins and RNA from macrophage cell membranes, exhibit a natural affinity for inflammatory targets.^53^ This inherent property enables them to evade rapid clearance by the mononuclear macrophage system in vivo, leading to increased accumulation at sites of osteonecrosis. Key targeting attributes, including cell adhesion molecules such as integrins, selectins, and chemokine receptors, particularly the elevated expression of macrophage-1 antigen (Mac-1) and RGD-binding integrins, are implicated in the effective “homing” to impairment locales.^54,55^ Next, some studies have revealed several M2-Exos function like modulating inflammation, promoting osteogenesis and angiogenesis.^56^ Jiao et al. suggested that M2-Exos can regulate neutrophil activation and NET formation through lipid mediator class switching.^57^ In this study, we introduced this innovative strategy for ONFH effective intervention and observed M2-Exos can regulate NETs-endothelial phenotype transition crosstalk in osteonecrosis area and thus prevent ONFH development. Furthermore, our investigation into the molecular mechanisms underlying M2-Exos treatment for osteonecrosis of the femoral head (ONFH) identified miR-93-5p as a potential key factor in the modulation of NETs formation and endothelial phenotype transition activation, ultimately preventing the development of ONFH. Wang et al. demonstrated that M2-Exos attenuated LPS-induced cell apoptosis by regulating the miR-93-5p/TLR4 axis. Chen et al. reported that miR-93-5p can provide protection effect against ischemia/reperfusion injury by regulating the TXNIP/NLRP3/Caspase-1 signaling pathway.^30,58^ Our study indicated novel function of M2-Exos miR-93-5p in perspective of NETs regulation which thus can serve as a potential ONFH treatment target.

## Materials and methods

### Clinical subchondral bone sample collection

Current study was approved by the Ethics Committee of the Second Affiliated Hospital of Xi’an Jiaotong University (Ethical Approval number No. 2019035). All tissue donors provided written informed consent for this study. Finally, subchondral bone samples from 3 FHF patients and 5 ONFH patients who underwent primary total hip arthroplasty were obtained from the Second Affiliated Hospital of Xi’an Jiaotong University. The subchondral bone tissue was washed once with DPBS, immersed in a mixture of 0.2% (w/v) collagenase type II (C6885, Sigma)/0.2% (w/v) dispase (D4693, Sigma), and placed on 37 °C horizontal rotators at 150 r/min for 4 h. The bone residue was removed with a 70 μm nylon filter after digestion, and the liquid portion was centrifuged at 400 × *g* for 5 min. The precipitate containing bone and bone marrow-derived cells and numerous small bone fragments was then resuspended in 20 mL of aMEM and placed in a 50 mL centrifuge tube containing 20 mL of a 75% (v/v) Percoll solution (P1644, Sigma). The whole mixture was centrifuged at 450 × *g* for 20 min. Then, approximately 5 mL of the nucleated cell layer was collected, diluted with aMEM (1:10), and recentrifuged at 400 × *g* for 5 min. Other samples were snap-frozen and stored in liquid nitrogen until further analysis.

### Single cell sequencing and high-throughput RNA sequencing

1. scRNA-seq: Single-cell suspension was adjusted to 1 × 10^5^ cells/mL in PBS and then loaded onto a microfluidic chip (GEXSCOPE Single Cell RNA-seq Kit, Singleron Biotechnologies) and scRNA-seq libraries were constructed according to the manufacturer’s instructions (Singleron Biotechnologies). The resulting scRNA-seq libraries were sequenced on an Illumina HiSeq X10 instrument with 150 bp paired end reads. Gene expression matrices were generated based on raw reads. Meanwhile, datasets SRR18163493-SRR18163503 were downloaded from SRA database.
2. subchondral bone sample RNA-seq: Frozen subchondral bone samples were rapidly ground in liquid nitrogen. Total RNA was extracted by TRIzol reagent (Invitrogen) following the manufacturer’s instructions. The NEBNext Poly(A) mRNA Magnetic Isolation Module (New England Biolabs), RiboZero Magnetic Gold Kit (Illumina), and KAPA Stranded RNA-Seq Library Prep Kit (Illumina) were used to carry out RNA enrichment and sequencing library generation. An Agilent Bioanalyzer 2100 system was used to conduct the quality control analysis. Finally, high-throughput RNA sequencing was performed based on the Illumina HiSeq 6000 sequencing platform (Illumina) using the TruSeq SR Cluster Kit (Illumina).

### Bioinformatics analyses

First, read one without polyT tails were filtered, then cell barcodes and unique molecular identifiers (UMI) were extracted. Adapters and polyA tails were trimmed before aligning read two to GRCh38 with ensemble version 92 gene annotation. Second, reads with the same cell barcode, UMI and gene were grouped together to count the number of UMIs per gene per cell. We removed cells that had either lower than 200 or higher than 5 000 expressed genes. Furthermore, we discarded cells with more than 30 000 UMIs and mitochondria content higher than 30%. We opted out the batch effect correction algorithm based on the highly consistent results among patients. Harmony was used as the batch effect removal method. We used Seurat to first normalize expression matrices by function NormalizeData and ScaleData. Then FindVariable function was applied to select the top 1 000 variable genes and perform principle component analysis. The first 20 principle components and resolution 0.8 were used with FindClusters function to generate 24 cell clusters. To assign one of the 10 major cell types to each cluster. CellChat package in R software were used to carried out the cell-cell communication analyses. Bulk RNA sequencing data were used to estimate the cellular composition of ONFH bone tissue based on CIBERSORT method.

### Construction of ONFH animal models

All animal experiments included in this study were approved by the Laboratory Animal Care Committee of Xi’an Jiaotong University. In this study, we established ONFH rat models and ONFH mouse model. A total of 80 adult SPF Sprague-Dawley rats and 40 C57BL/6 J mice were purchased from the Medical School of Xi’an Jiaotong University Animal Center. (1) TONFH rat modeling: the rats were anesthetized with sodium pentobarbital (50 mg/kg, intraperitoneal injection), the fur was removed, and the skin was sterilized around the surgical area. An incision was made in the hip joint (right side) and then separated by layer to expose the femoral head. The hip joint was dislocated, the round ligament was removed, blood supply to the femoral head was stopped, the wound was disinfected, and then the incision layer was replaced. All the rats were sacrificed for femoral heads after 6 weeks. (2) SONFH rat modeling: intraperitoneal (i.p.) injection of LPS (20 μg/kg, Sigma-Aldrich) was given for the first day. Then an intramuscular (i.m.) dose of methylprednisolone (MPS, 60 mg/kg, Pfizer) was administered each day for the next 3 days. MP was injected into the left and right gluteus muscles alternately. Animals were sacrificed for femoral heads 6 weeks after MP medication. (3) Adeno-associated virus-treatment (AAV-treatment) rat modeling: the rats were anesthetized with sodium pentobarbital (50 mg/kg, intraperitoneal injection), the fur was removed, and the skin was sterilized around the surgical area. An incision was made in the hip joint (right side) and then separated by layer to expose the femoral head. The hip joint was dislocated, the round ligament was removed, blood supply to the femoral head was stopped. Carefully sutured the joint capsule and injected AAV to joint capsule. Then the wound was disinfected, and then the incision layer was replaced. All the rats were sacrificed for femoral heads after 6 weeks. (4) SONFH mouse modeling: i.m. injection of MPS (Pfizer 25 mg/kg/d) was given for the first three weeks (injected on the day 1 to day 3 and rested on day 4 to day 7). Then the mice were rested for another three weeks. The administration schedule was total six weeks and animals were sacrificed for femoral heads.

### M2-Exos preparation

Mouse bone marrow was collected by rinsing the femur of C57BL/6 J mice (8–10 weeks) with a cold RPMI1640 medium (Procell, China) and red blood cells (RBCs) were lysed with RBCs lysis buffer (Thermo Fisher Scientific, USA). Macrophage M2 polarization was induced using 25 ng/mL murine macrophage colony-stimulating factor (M-CSF) (R&D Systems, USA), 20 ng/mL interleukin 4 (IL-4) (R&D Systems, USA) and 20 ng/mL interleukin-13 (IL-13) (R&D Systems, USA). The M2 polarization of BMMs were validated by flow cytometry of CD206 (BD biosciences, USA). After reaching 80% confluence, the cells were washed with PBS and incubated in a fresh complete medium containing exosome-depleted FBS (BI, China) for 48 h. The culture medium was collected and then centrifuged to remove cell debris. The resulting supernatant was filtered using a 0.22 µm filter (Millipore, USA) and concentrated using an ultrafiltration tube (Millipore, USA). Exoquick TC (System Biosciences, Bay Area, California, USA) was added to the concentrated supernatant and incubated for 12 h according to the manufacturer’s instructions. TEM was used to examined the diameter and microscopic morphology of extracted exosomes.

### Western blot assay

Total proteins from cells or exosomes were extracted using PMSF: RIPA (1:100) (Servicebio, China) and a BCA protein analysis kit (New Cell & Molecular Biotech Co., Ltd, China) was used to detect the total protein concentration. Mixed with 5×SDS loading buffer (Beyotime, China) thoroughly, the samples were heated at 95 °C for 10 min. Next, 20 µg of the protein was loaded onto a 10% SDS polyacrylamide gel and subjected to electrophoresis (100 V, 90 min). Then the proteins were transferred onto a PVDF membrane (400 mA, 20 min) (Millipore, USA) and blocked with TBS (Servicebio, China) containing 5% bovine serum albumin (BSA, BioFroxx, China). The PVDF membrane was then incubated with primary antibodies against CD63, TSG101, calnexin, α-SMA, TGF-β, VEGFA, PTEN, Akt (Abcam, UK) overnight at 4 °C. The following day, the PVDF membrane was incubated with HRP binding antibody (Abcam, UK) at room temperature for 90 min, and the signal was detected using ChemiDoc XRS (Bio-Rad, USA).

### Immunofluorescence staining

Cells fixed with 4% paraformaldehyde or bone tissue sections were permeabilized with 0.1% Triton X 100, and blocked with 1% BSA, the cells were then incubated overnight with primary antibodies against corresponding targets like cit-H3, MPO and so on (Abcam, UK) at 4 °C. After washing three times with PBST (Servicebio, China), the cells were incubated with fluorescent secondary antibodies (Abcam, UK) at room temperature for 1 h. Finally, DAPI were used to perform nuclear staining.

### Isolation of neutrophils, NETs formation and Collection

Neutrophils were isolated from human whole blood by Percoll density gradient centrifugation. The isolated neutrophils were seeded into a 24-well plate and treated with 100 nmol/L PMA (P1585, Sigma, USA) for 4 h to activated the NETs formation. Adherent NETs were washed with prewarmed PBS and isolated by partially digesting the NETs in fresh DMEM/F12 medium supplemented with 5 U/mL micrococcal nuclease (MNase). DNA content was measured using the Quantifluor dsDNA kit in black 96-well plates using serially diluted lambda DNA as a calibration standard. NETs were stored at –80 °C until further use.

### ROS level detection

HUVECs were seeded in 96-well plates and divided into control group, NETs treated group and M2-Exos rescue group. After the HUVECs had adhered to the plate surface, NETs treated group and M2-Exos rescue group were treated with NETs. Meanwhile, in the M2-Exos rescue group, M2-Exos were added at a concentration of 300 µg/mL. After 48 h, each group was then incubated with 10 μmol/L DCFH-DA at 37 °C for 1 h according to the instructions of the Reactive oxygen species staining kit (Beyotime, China). Fluorescence microscopy (Leica, Germany) was used to detect ROS levels.

### Tube formation assay

A precooled 96-well cell culture plate was coated with matrix gel (356234, Corning) applied at 50 µL per well and placed in an incubator at 37 °C for 1 h. Thereafter, HUVECs was added to each gel-coated well. After 6 h of incubation at 37 °C under 5% CO_2_. Finally, the results were observed under a microscope. The branch points and total tube length in each image were automatically counted using the Angiogenesis Analyzer plugin in ImageJ software.

### HUVECs transwell migration assay

An 8 μm pore size 24-well transwell insert (Corning Costar, USA) was used to perform HUVECs transwell migration assay. 500 μL of medium containing 20% FBS was added to the lower chamber, and 200 μL of HUVECs suspension containing 1% FBS was seeded into the upper chamber at a density of 1 × 10^5^ cells/well. After being incubated for 12 h, the nonmigratory cells on the upper surface of the membrane were gently removed with cotton swabs. The migrated cells on the lower surface of the membrane were fixed with 4% paraformaldehyde and stained with 0.1% gentian violet.

### Statistics

Data are expressed as mean ± SD. Student’s *t* tests, Mann–Whitney U tests were carried out for the difference analysis of continuous variables as appropriate. Statistical significance was considered at a two-sided *P* value < 0.05 for all analyses.

## Supplementary information

Supplementary Information

## Data Availability

For original data, please contact the corresponding author Prof. Pei Yang by sending an e-mail to yangpei@mail.xjtu.edu.cn.
