# Supplementary material for: M2 macrophages-derived exosomes for osteonecrosis of femoral head treatment: modulating neutrophil extracellular traps formation and endothelial phenotype transition
Source: Bone Res. 2025 Apr 1;13:42. doi: 10.1038/s41413-025-00412-5 (PMC11961764; doi:10.1038/s41413-025-00412-5)
Supplement: Supplementary file 1 — Supplementary Information [file 41413_2025_412_MOESM1_ESM.docx]

Supplementary Information


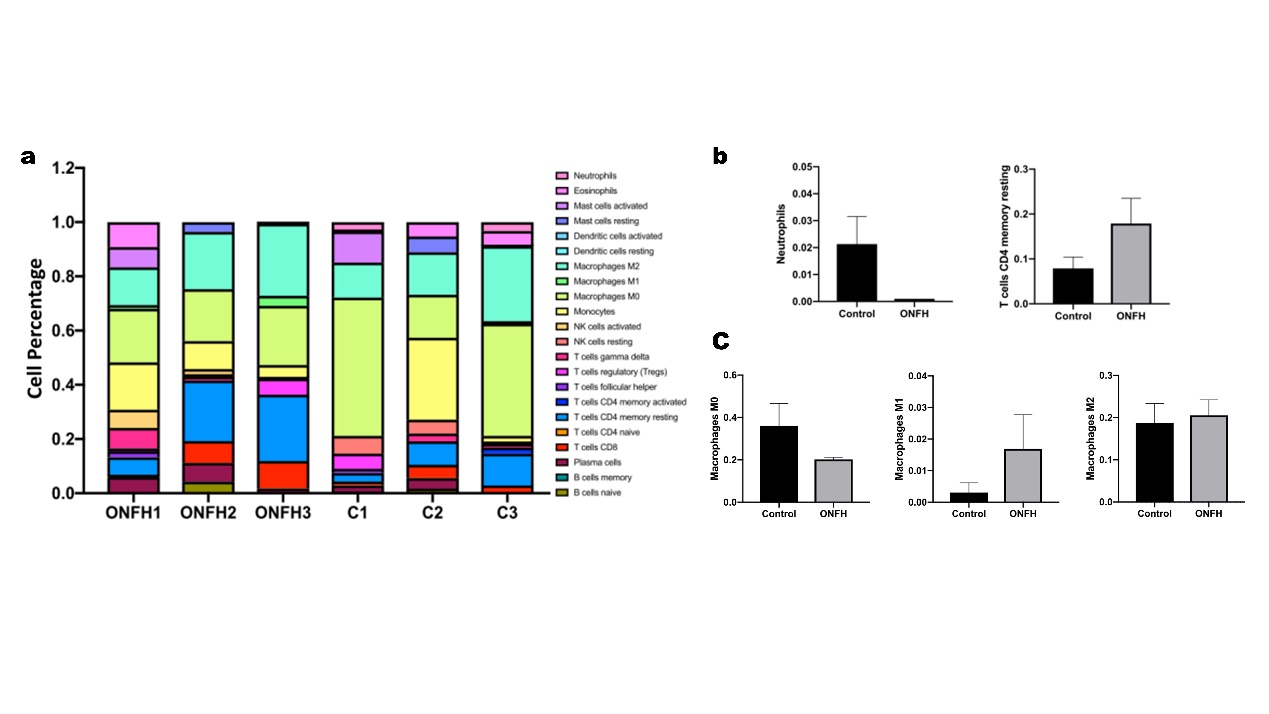


**Supplementary figure 1** ONFH bulk RNA-seq cellular composition analyses. a) bulk RNA-seq cellular composition analyses for three ONFH bone samples and three control group samples based on CIBERSORT. b) ONFH-associated significant cell clusters composition analyses including neutrophil and T cells CD4 memory resting. c) ONFH-associated M0, M1 and M2 macrophage cluster composition analyses.


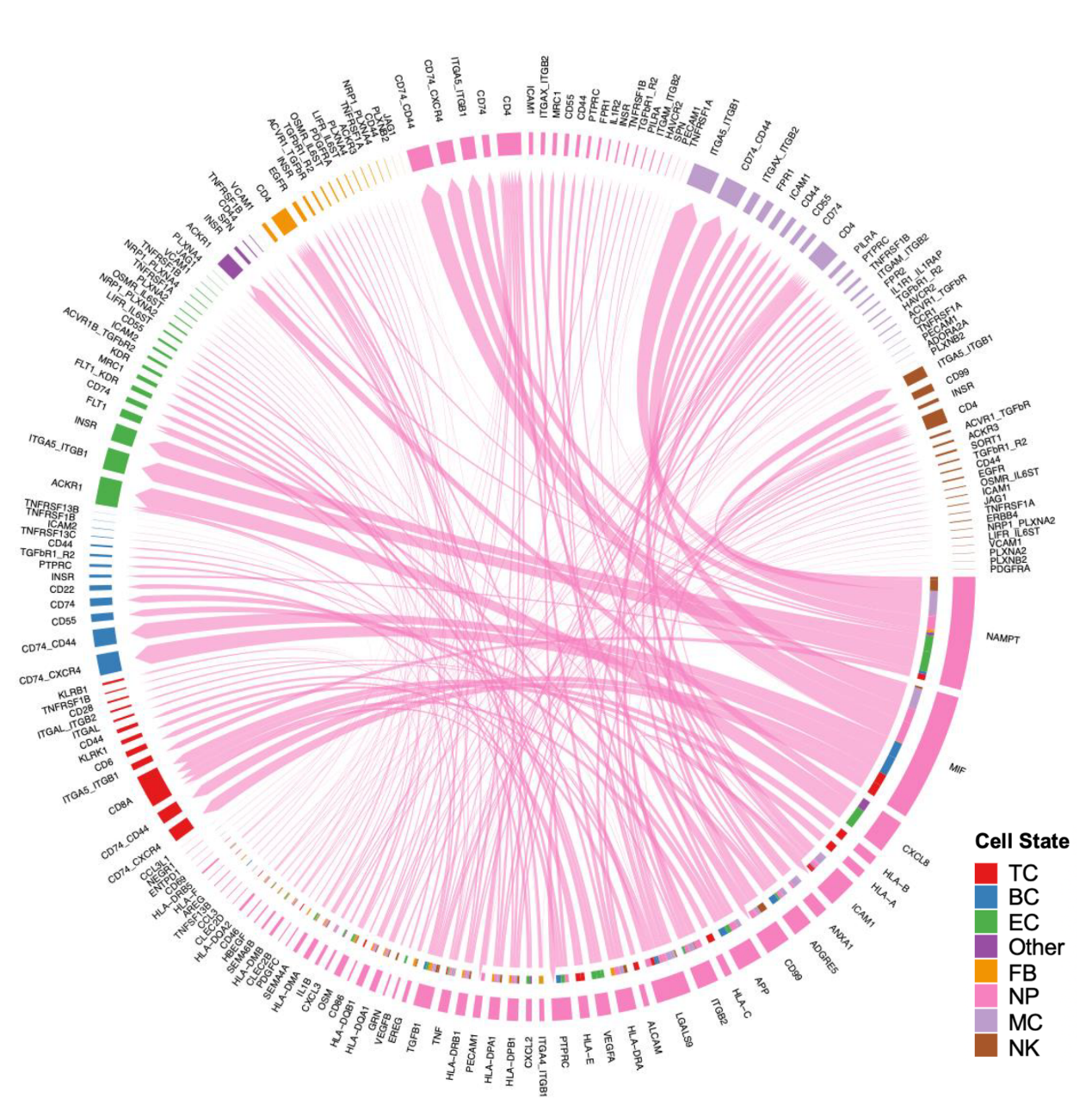


**Supplementary figure 2** Hip osteoarthritis group cell-cell communication analysis.


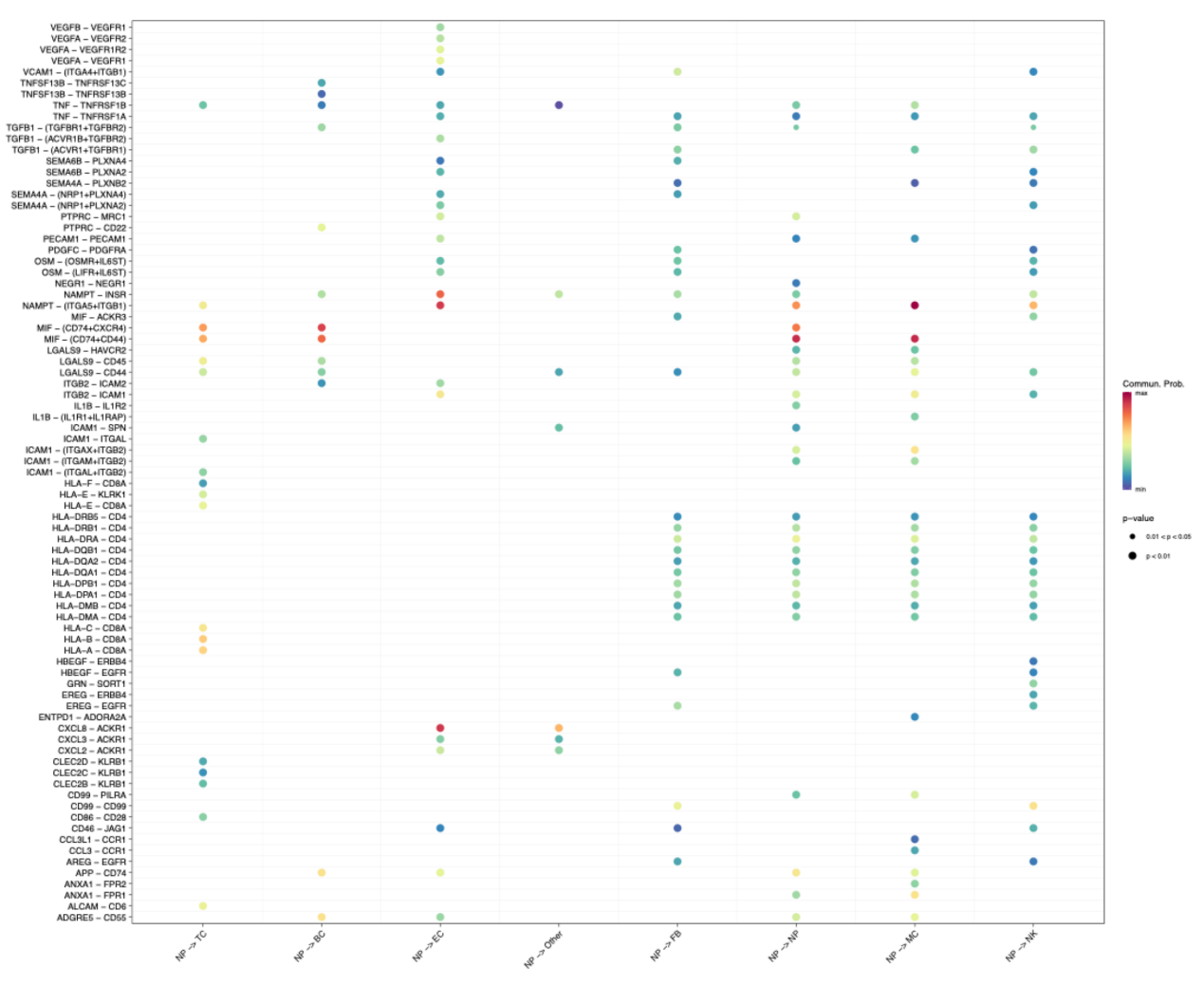


**Supplementary figure 3** Hip osteoarthritis group cell-cell communication dot plot.


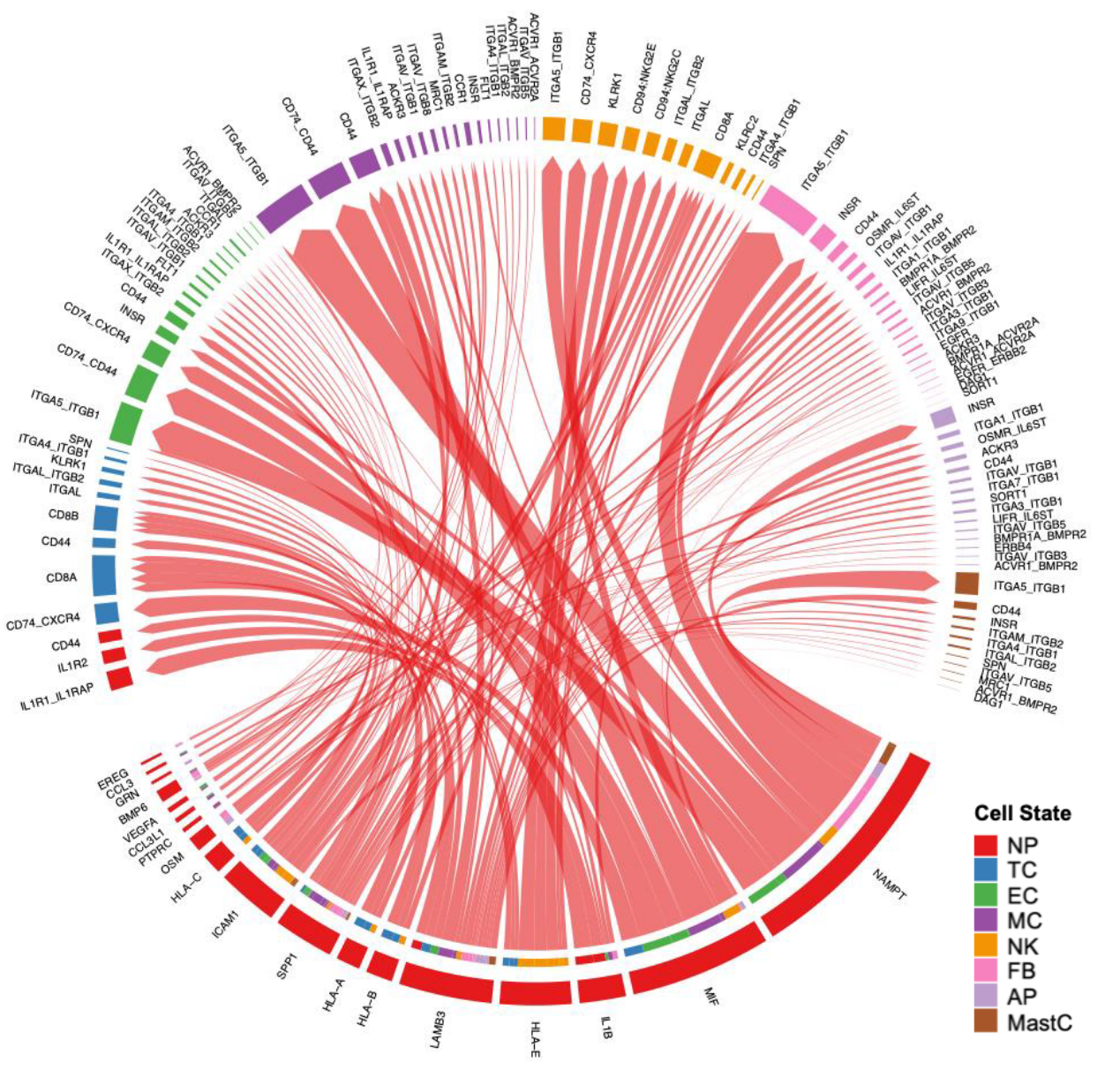


**Supplementary figure 4** Femoral neck fracture group cell-cell communication analysis.


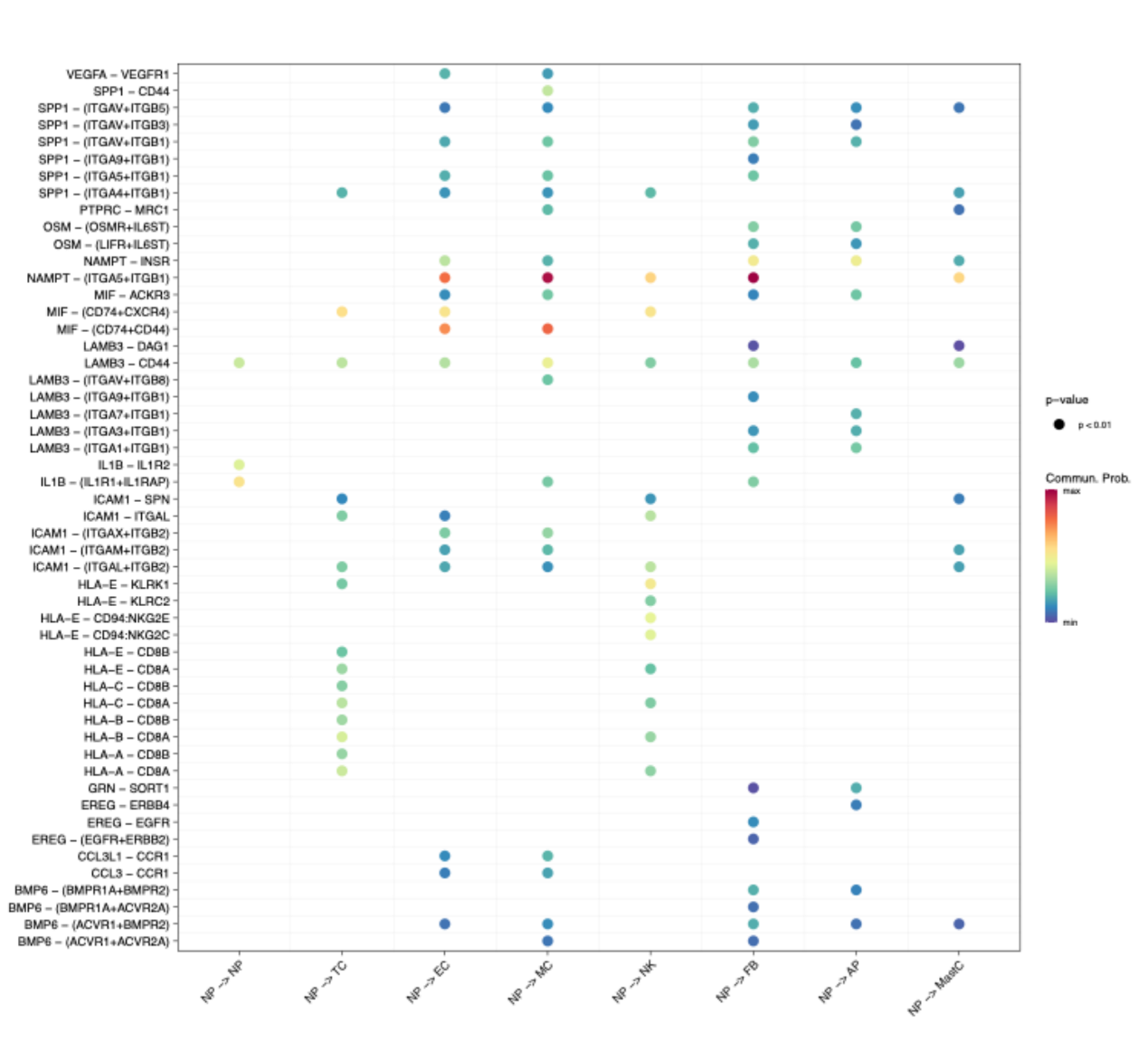


**Supplementary figure 5** Femoral neck fracture group cell-cell communication dot plot.


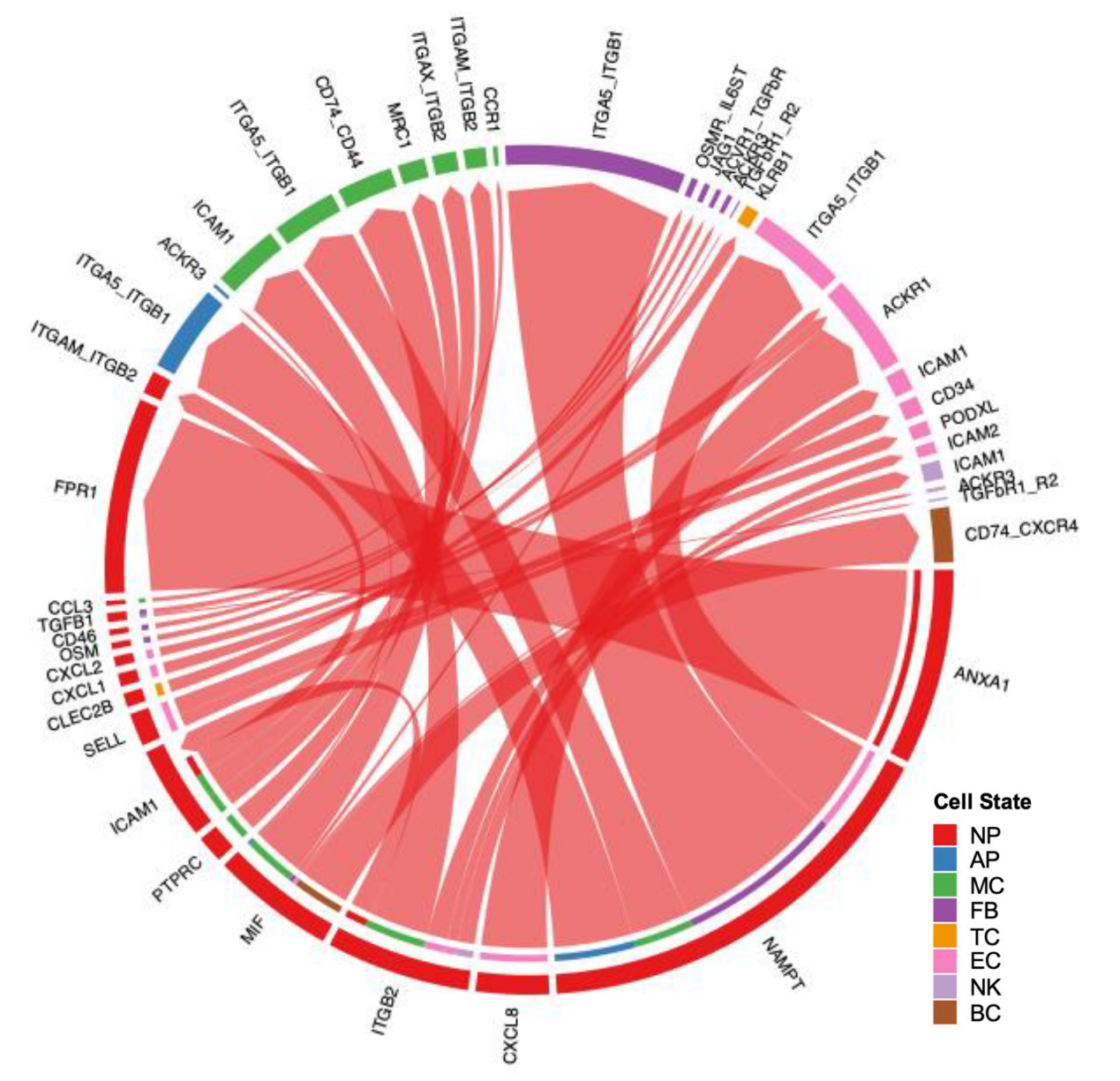


**Supplementary figure 6** ONFH group cell-cell communication analysis.


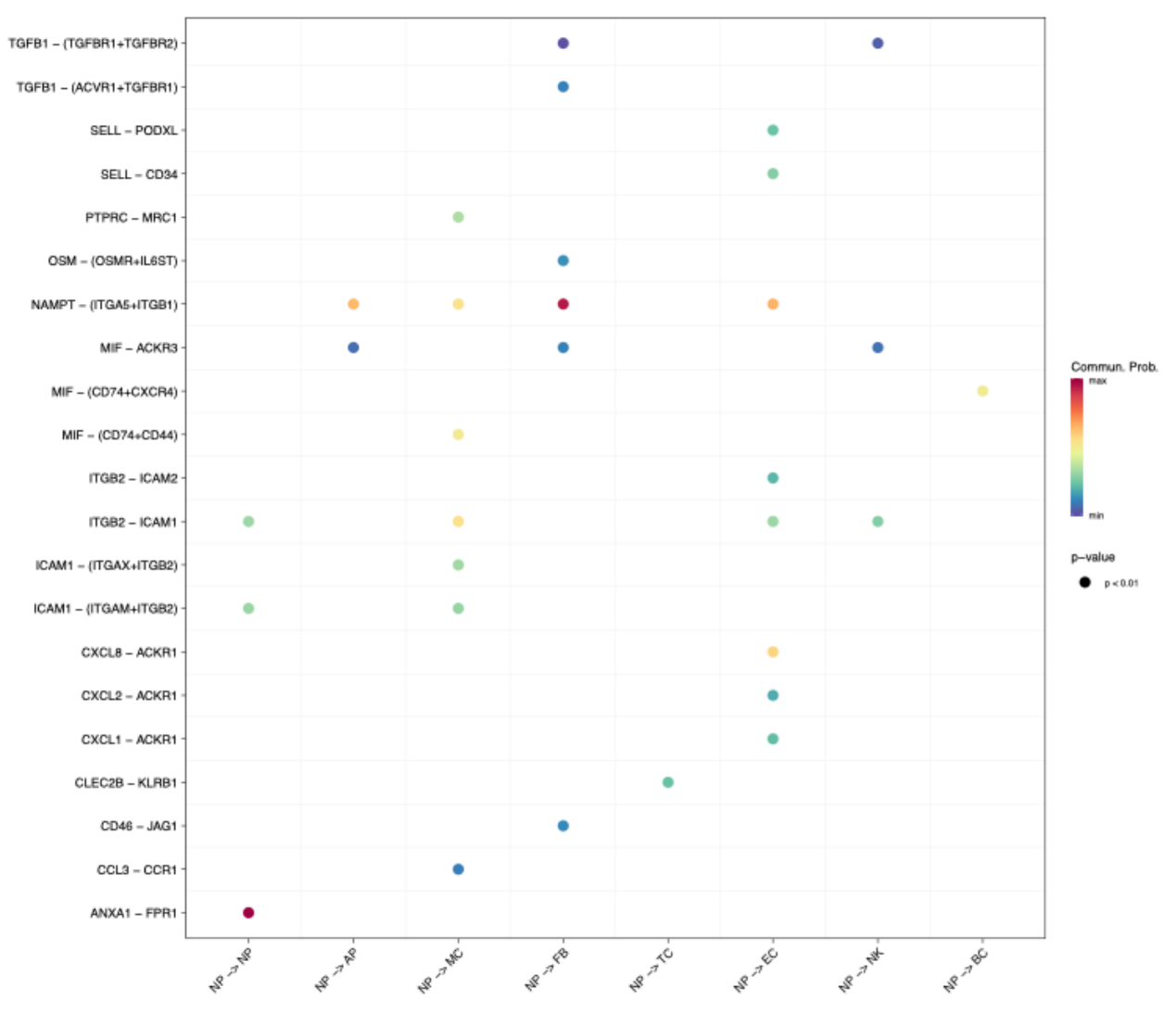


**Supplementary figure 7** ONFH group cell-cell communication dot plot.


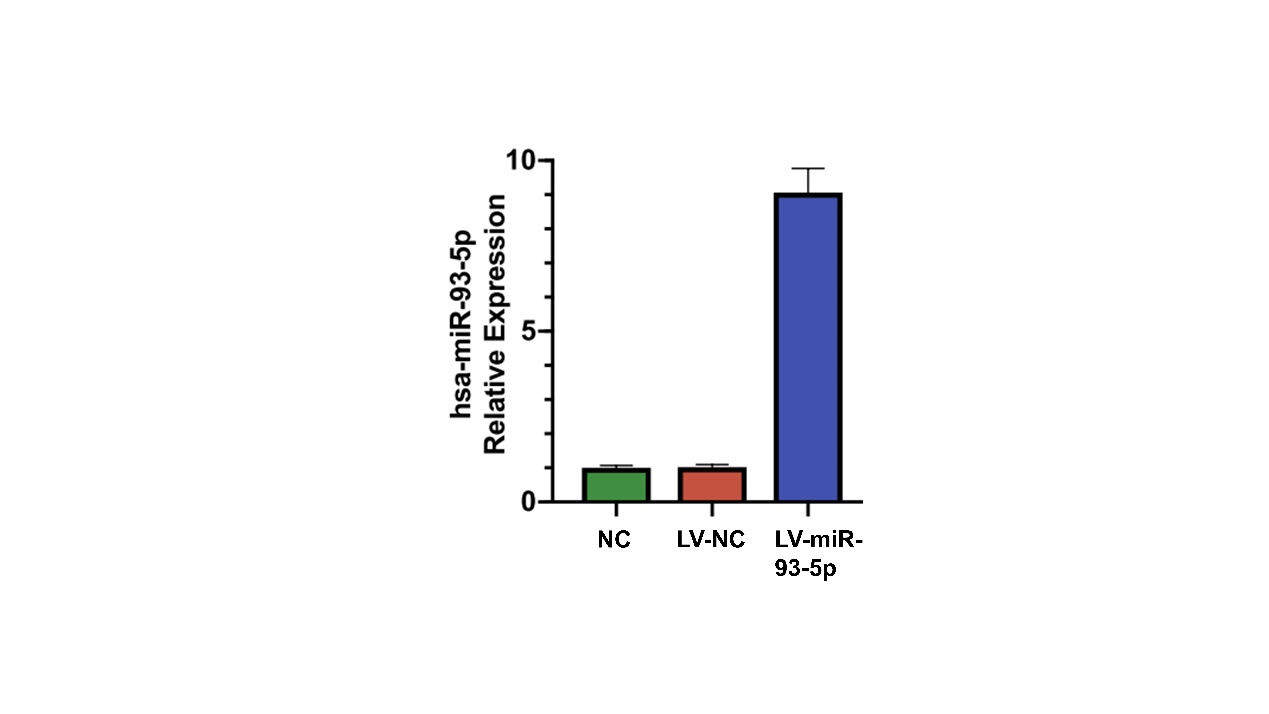


**Supplementary figure 8** RT-qPCR assay for has-miR-93-5p expression levels of negative control group, lentiviral negative control group and lentiviral-miR-93-5p group in HUVECs.


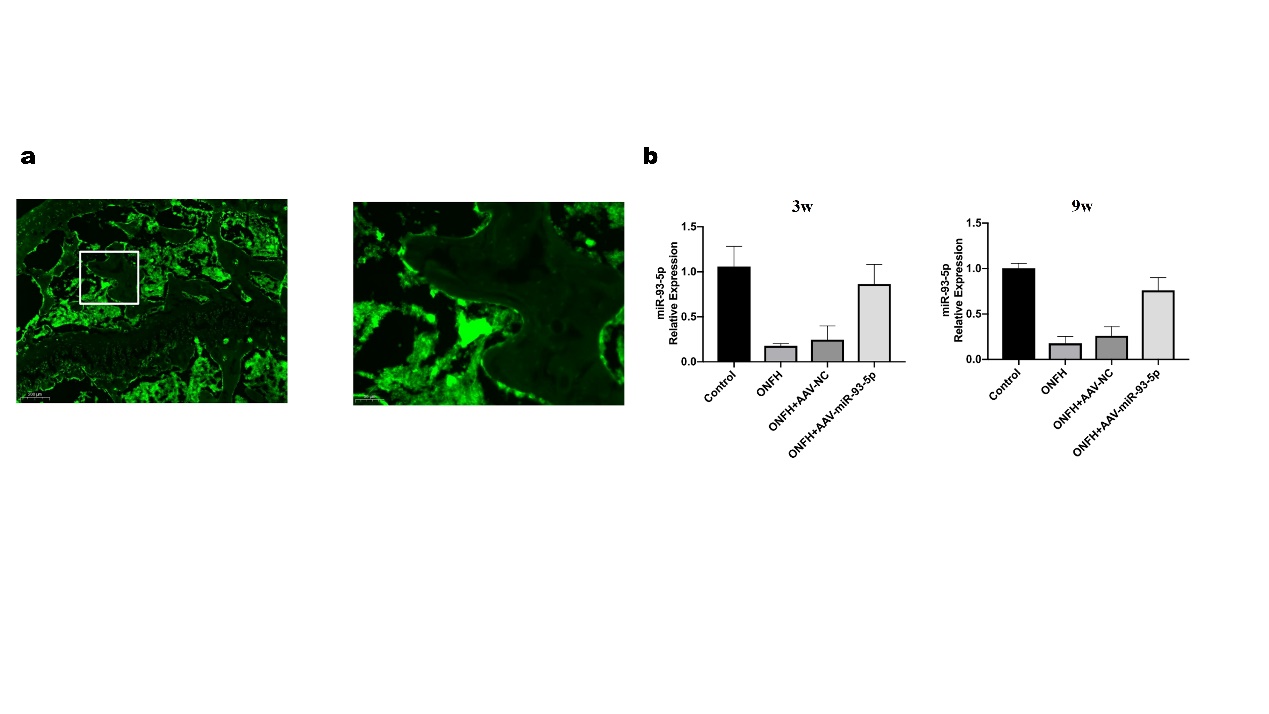


**Supplementary figure 9** a) ONFH rat model femoral head fluorescence pictures of green fluorescent protein (GFP) expression after injection of GFP-labeled AAV9 for 6 weeks. b) RT-qPCR assays of miR-93-5p expression levels after injection of AAV9 for 3 and 9 weeks so as to confirm the period of validity.


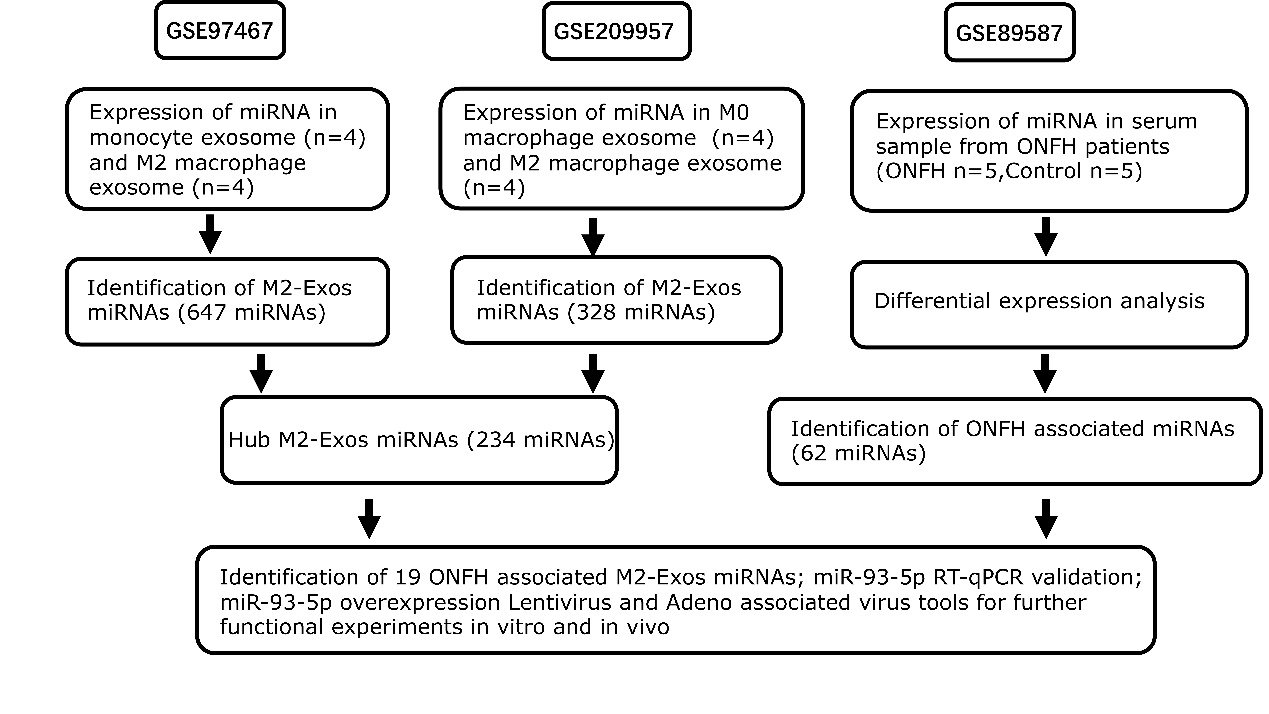


**Supplementary figure 10** MiR-93-5p identification flowchart.


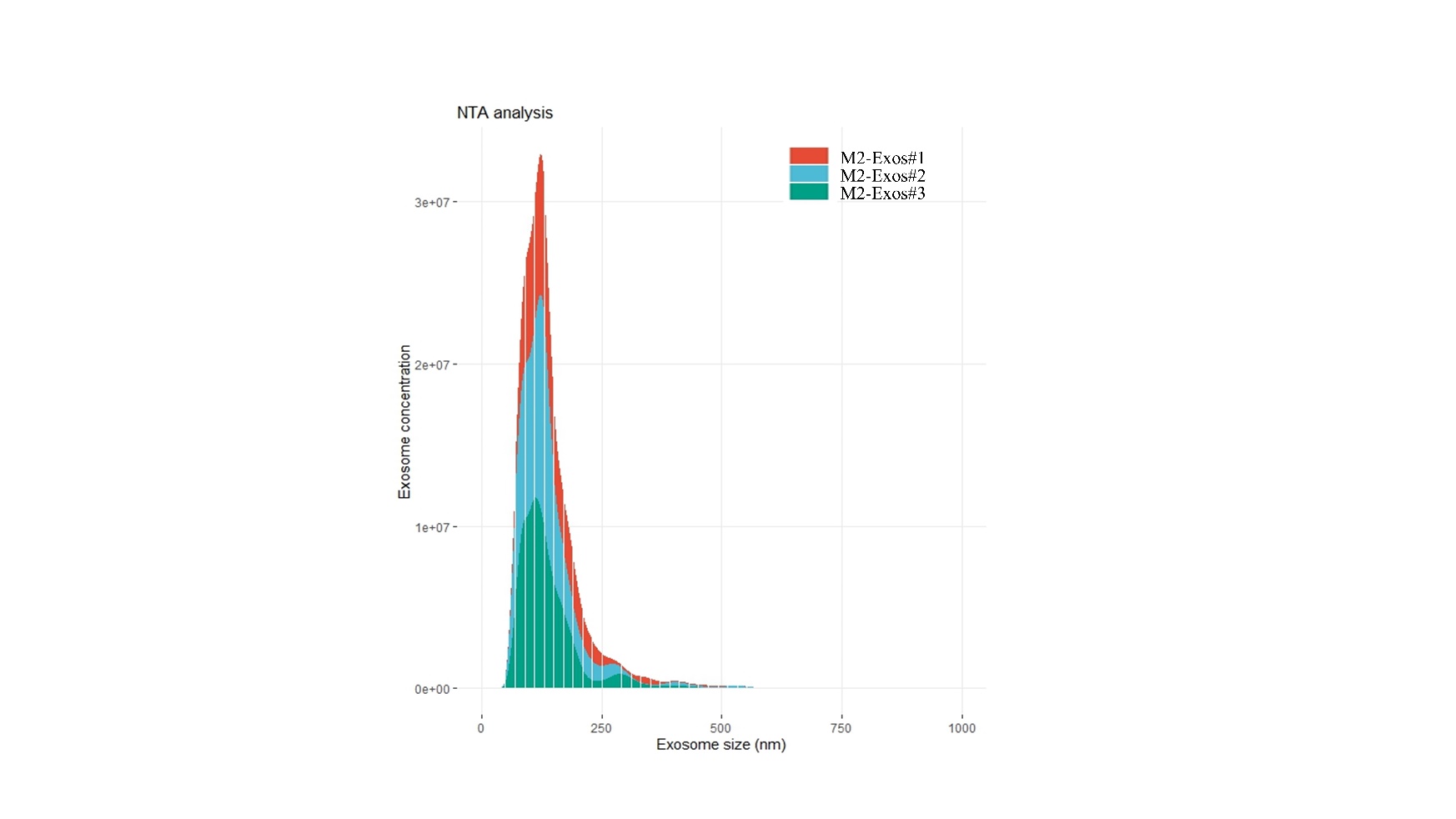


**Supplementary figure 11** M2-Exos nanoparticle tracking analysis


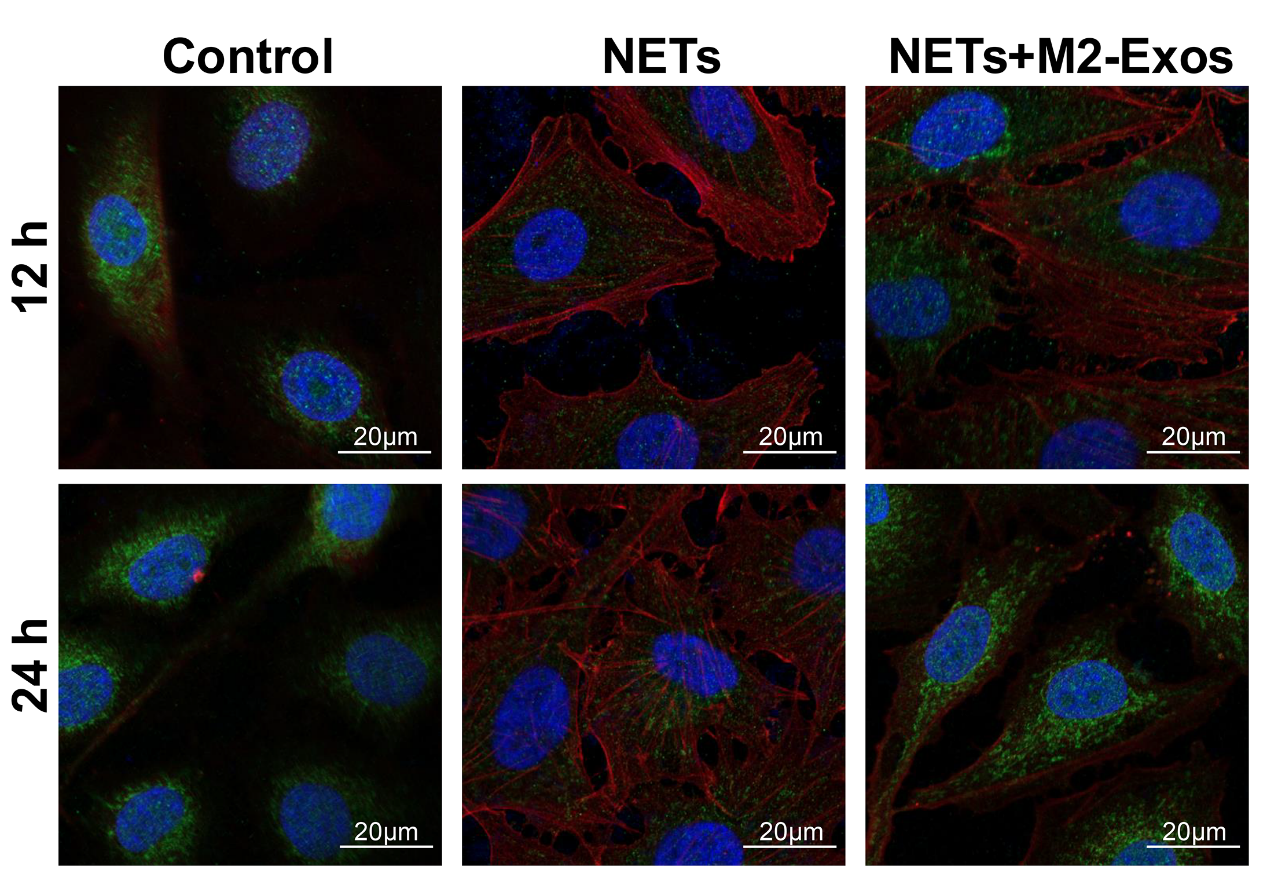


**Supplementary figure 12** Immunofluorescent staining of NETs and M2-Exos treated HUVECs (α-SMA: red; CD31: green; DAPI: blue).

**Supplementary table 1 Primer sequences for target genes**

| Gene | Primer sequence |
| --- | --- |
| hsa-miR-93-5p | Forward-5’- CAAAGTGCTGTTCGTGCAGGTAG-3’ |
| Human-RNU6 | Forward-5’- AGAGAAGATTAGCATGGCCCCT-3’ |
| rno-miR-93-5p | Forward-5’- CAAAGTGCTGTTCGTGCAGGTA -3’ |
| Rat-RNU6 | Forward-5’- AGAGAAGATTAGCATGGCCCC -3’ |
